# Gender Differences in Self-Regulated Online Learning During the COVID-19 Lockdown

**DOI:** 10.3389/fpsyg.2021.752131

**Published:** 2021-09-16

**Authors:** Xiaohong Liu, Wei He, Li Zhao, Jon-Chao Hong

**Affiliations:** ^1^School of Educational Science, Nanjing Normal University, Nanjing, China; ^2^Department of Industrial Education, Institute for Research Excellence in Learning Sciences, National Taiwan Normal University, Taipei City, Taiwan

**Keywords:** self-regulated, online learning, gender difference, high school students, COVID-19

## Abstract

Due to the spread of the epidemic around the world, online learning has received greater attention. Self-regulated learning (SRL) is an important factor for students to achieve academic success. This study investigated the gender differences in SRL and three sub-constructs of SRL in the context of online learning, that is the preparatory, performance, and appraisal phases. A total of 400 high school students (males = 125, females = 275) from China participated in this study. In order to identify whether there were gender differences in their self-regulated online learning (SROL), independent sample *t*-test was performed. The results showed that there were significant gender differences in the SROL (*t* = –3.334, *p* = 0.001 < 0.01, *d* = –0.410) and the three sub-constructs of SROL (preparatory: *t* = –0.702, *p* = 0.008 < 0.01, *d* = 0.018; performance: *t* = –3.801, *p* = 0.000 < 0.01, *d* = 0.456; appraisal: *t* = –3.120, *p* = 0.002 < 0.01, *d* = 0.361). The findings indicated that females performed better than males in all three dimensions of learners’ online self-regulated learning.

## Introduction

The COVID-19 epidemic has spread around the whole world. In the face of the sudden outbreak of the epidemic, offline education sites have been closed, and learners have been forced to accept online learning at home. Online learning can better accommodate students’ diverse needs (Zhu et al., 2020; Zhao et al., 2021a,b), but it can also result in learners experiencing a sense of isolation (Barak et al., 2016). Some surveys (e.g., Sawarkar et al., 2020; Velichová et al., 2020) during the pandemic have found that students do not agree that online learning is better than offline learning at school. In the online learning environment, students are subject to less supervision from teachers and peers, and there are fewer opportunities for interaction and feedback between teachers and students, which may lead to some more obvious negative learning consequences such as procrastination and inattention (Cheng and Xie, 2021; Hong et al., 2021). When transitioning from face to face learning to a full online learning environment, students need to use appropriate strategies to ensure that their online learning is effective. One of the appropriate strategies is self-regulated learning (SRL) (Adam et al., 2017), which refers to the extent to which learners actively engage in the learning process, and thus has an important impact on academic success (Zimmerman, 2000). Pintrich (2004) highlighted that the SRL model is an individual’s capability to self-regulate, and is influenced by environmental and personal factors. Moreover, students’ SRL is developed based on motivational beliefs and mediates the relationship between online learning environmental and personal characteristics (Cheng and Xie, 2021). Accordingly, there is a need to further explore the self-regulation of high school students in online learning environments.

In online learning environments, students can regulate their personal functions through using self-regulation related strategies, to benefit from the online learning. Thus, the change in learning environment requires learners to engage in SRL to ensure that they can adapt to online learning as soon as possible (Adam et al., 2017). Online learning environments require learners to have higher self-regulation learning ability related characteristics, especially during the COVID-19 lockdown (Zhu et al., 2020; Hong et al., 2021). Regarding gender as a personal characteristic, Wu and Cheng (2019) examined who is better adapted to learning online within the personal learning environment. They found that males adopted more behavioral strategies than females to deal with their disorientation during online learning. Another study indicated that in Pakistan, females have better online communication self-efficacy than males during COVID-19 online learning (Rafique et al., 2021). To address the inconsistency in the research results, more empirical research is required in order to be able to draw firm conclusions about gender differences in SRL in online learning contexts. However, few studies have focused on how gender affects these three phases. Thus, the present study explored the gender differences in the phases of SRL (i.e., the preparatory, performance, and appraisal phases, Adam et al., 2017) among high school students in the online learning context during the COVID-19 lockdown.

Thus, more empirical research is required in order to be able to draw firm conclusions about gender differences in SRL in online learning contexts. Moreover, researchers have discussed many models and phases of SRL. In general terms, the SRL model comprises the preparatory, performance, and appraisal phases (Adam et al., 2017). However, few studies have focused on how gender affects these three phases. In order to fill this gap, we explored the gender differences in the phases of SRL among high school students in the online learning context during the COVID-19 lockdown.

## Theoretical Background

### Self-Regulated Learning

Self-regulated learning (SRL) was defined as “an active, constructive process whereby learners set goals for their learning and then attempt to monitor, regulate, and control their cognition, motivation, and behavior, guided and constrained by their goals and the contextual features of the environment” (Pintrich, 2000, p. 453). Thus, there is an association between SRL and learners’ academic achievement (Ergen and Kanadli, 2017) and SRL has been recognized as a key competency; the education system should therefore make every effort to ensure that students can develop SRL (Granberg et al., 2021). SRL could be described as a multi-component process (Zimmerman and Risemberg, 1997). It involves metacognitive, cognitive, and motivational processes (Heirweg et al., 2019). The metacognitive component refers to allowing the higher order thinking processes of planning, monitoring, and evaluating learning (Zimmerman, 2000). The cognitive component encompasses learning strategies that process information, such as rehearsal, summarization, paraphrasing, etc. (Zimmerman, 2000). The motivational component includes learners’ extrinsic and intrinsic motives for learning, and the effort and persistence that they exert, including positive self-talk, positive activities, making tasks more interesting, etc. (Pintrich, 2004).

SRL was also defined as a cyclical process which involves a number of different phases (Heirweg et al., 2019). SRL models comprising different constructs have been developed (e.g., Irvine et al., 2020; Pintrich, 2000; Zimmerman, 2000). For example, Pintrich’s (2000) SRL model explains different aspects of SRL in accordance with the four phases: Namely the forethought, planning, and activation; monitoring; control and reaction; and reflection phases. Zimmerman (2000) described self-regulation as a cyclical process consisting of three phases: forethought, performance, and self-reflection, whereas Li et al. (2020) clustered SRL as forethought, adaptive, and monitoring self-regulated behavior. These SRL models share similar elements and processes (Chen and Bonner, 2020). Basically, SRL has been conceptualized as comprising a three-phase process, including the preparatory, the performance, and the appraisal phases (Adam et al., 2017). Many studies have explored the learner’ SRL. For example, Maison and Syamsurizal (2019) analyzed the correlations between learning environments and self-regulation in learning physics. Lee et al. (2020) examined the massive open online course learners’ task value of SRL strategies, and Hong et al. (2021) explored the relationship between procrastination and SRL, but few studies have focused simultaneously on all three of these phases (Zeidner and Stoeger, 2019).

In online learning environments, learners’ self-regulating ability is a key factor in overcoming the obstacles in the preparatory, performance, and appraisal phases. In the preparatory phase, students need to consider various external factors, such as whether the network is stable when accessing the computer at home, and regulating their emotions to avoid distractions during the online lessons (Hong et al., 2021). In terms of the performance phase, students must be more active in using task strategies and monitoring how much time they spend on each lesson compared to dedicated class hours. In the appraisal phase, students face higher barriers to seeking help (Aleven et al., 2015), and still need to evaluate and adjust their progress. The closure of campuses due to the COVID-19 pandemic may have a negative impact on students’ SRL by imposing higher requirements on students’ preparation, grades, and evaluation. Considering this, this study employed the (a) preparatory, (b) performance, and (c) appraisal phases as a cyclic process to explore the three phases of high school students’ self-regulated online learning (SROL).

### Three Phases of SROL

Adequate preparation in advance can make things get twice the result with half the effort. Preparedness is fundamental to the successful role of management practices during outbreaks of COVID-19 (Sotomayor-Castillo et al., 2021). Therefore, Hong et al. (2021) focused on students’ self-regulated behavior prior to taking part in online learning, and specified the preparatory phase including mood adjustment and structuring the environment before participating in online lessons during COVID-19. Thus, this study used mood adjustment and environmental structuring as components of the preparatory phase. Mood adjustment refers to adjusting emotions before online lessons to activate pre-reflection and to avoid being distracted during online lessons (Hong et al., 2021). Environmental structuring refers to checking and adjusting the surrounding learning environment to make it suitable for online lessons before online lessons (Martinez-Lopez et al., 2017).

Pintrich (2000) highlighted that the second stage of SRL involved an active, constructive process to optimize performance and effectively manage learners’ time and effort. During the performance phase, learners adapt time and specific strategies to complete tasks. Accordingly, Hong et al. (2021) specified that the performance phase focuses on task strategy and time management while participating in online lessons during the COVID-19 lockdown. Thus, this study used task strategy and time management as components of the performance phase. Task strategy refers to the arrangement of learning tasks and activities during online lessons in order to control their emotions, attention, or environment (Ridgley et al., 2020), and to ensure that the learning process, learning outcomes, and the effort exerted in learning are maximized (Effeney et al., 2013). Time management refers to structuring, protecting, and adapting their time to changing conditions during online lessons (Aeon and Aguinis, 2017).

After learning tasks are completed, learners monitor their progress, reflect on their learning effectiveness (Zimmerman, 1990; Cleary et al., 2012), and develop help-seeking schemes to improve their learning processes (Zimmerman, 2000). The appraisal phase is important for adjusting and guiding the next round of learning. Hong et al. (2021) specified that the appraisal phase focuses on self-evaluation and help-seeking after participating in online lessons during the COVID-19 lockdown. Thus, this study used self-evaluation and help-seeking as components of the appraisal phase. Self-evaluation refers to actively monitoring and judging each learner’s particular progress and performance according to their personal learning goals after online lessons (Schunk, 2005; Bound, 2013). Help seeking refers to seeking aid from student peers, information resources, or a teacher after online lessons (Aleven et al., 2006).

### Gender in SROL

Many studies have examined student gender differences in SRL with respect to several components. In an early study, Zimmerman and Martinez-Pons (1990) found that girls had a far greater tendency than boys to employ the strategies of self-monitoring, goal setting, planning, and structuring of their study environment, while in Bidjerano’s (2005) review study, female students showed better ability than males in using self-regulated strategies including information organization, metacognition, time management skills, elaboration, and effort. Hargittai and Shafer (2006) found that females tended to assess their own skills significantly lower than males evaluated theirs, while Özsoy-Güneş et al. (2014) found that the average self-regulation scores of their female participants were significantly higher than those of the males in the areas of “planning and determining aims” and “lack of self-direction.” According to these studies, there have been conflicting findings regarding gender and the dependent components of SRL (Martinez-Lopez et al., 2017; Stanikzai, 2019). The gender difference in the three phases (the preparatory phase, the performance phase, and the appraisal phase) has not been discussed in the context of the COVID-19 lockdown (Alghamdi et al., 2020). Hong et al. (2021) proposed that further research on gender difference in the context of the COVID-19 lockdown is required. Thus, the present study focused on exploring gender differences in high school students’ practice of SROL lessons during the COVID-19 lockdown.

### Research Hypotheses

In school, many students have difficulty regulating their learning and although they may try, their strategies may be unproductive, they may not be able to adjust them to the learning task or situation, or they may not lead to deep-level learning processes (Perry et al., 2004; Pintrich, 2004; Winne, 2005). However, research indicates that individual background characteristics can lead to differences in SRL. For example, gender can contribute to differences, with boys tending to show less self-regulating behavior than girls (Pajares, 2002; Vandevelde et al., 2013). A comparison of gender differences in the frequency of students’ SRL profiles indicated that girls reported more favorable SRL profiles than boys (Heirweg et al., 2019). Another study indicated that female students reported that they adopted more goal setting and more environment structuring strategies than male students in Massive Open Online Courses (MOOCs) (Li, 2019). A more person-oriented approach is therefore required for online learning during the COVID-19 lockdown (Alghamdi et al., 2020), but how gender influences differences in individuals’ application of SROL components has not been extensively studied. Thus, this SROL approach was adapted by analyzing gender profiles in an online learning context. In order to explore gender differences among high school students’ three phases of SROL during the COVID-19 lockdown, the following three hypotheses were proposed in this study:

H1: There are gender differences in the Preparatory phase of SROL.

H2: There are gender differences in the Performance phase of SROL.

H3: There are gender differences in the Appraisal phase of SROL.

## Materials and Methods

### Sample and Procedure

Most of the previous studies on gender differences in self-regulation were carried out in universities (Virtanen and Nevgi, 2010; Park, 2019; Wong et al., 2019; Lee et al., 2020). In contrast, there has been little research involving high school students. Thus, the present study used purposive sampling to target high school students who had enrolled in online courses for two semesters in China. Questionnaires were delivered to social networks via a web-based survey system. A message was sent requesting faculty members of different high schools to distribute the questionnaire to their students in online courses during the coronavirus outbreak. A total of 425 students took part in the online survey voluntarily and anonymously. Incomplete questionnaires were removed and 400 samples were retained for analysis, giving a 94.1% useful return rate.

The participants consisted of 125 males (31.3%) and 275 females (68.7%). The first-grade students accounted for 145 (36.3%), second grade students for 116 (29.0%) and third grade students for 139 (34.7%). Ages ranged from 15 to 22 years (*M* = 16.9). Additionally, all the participants took online courses. Their average number of online learning hours per day was 2.25. The number of online courses in the semester was between 2 and 9 (*M* = 4.79). Most of the participants (Frequency = 378, 94.5%) took courses online up to 50% of the time during the two semesters.

### Instruments

The items in the questionnaire were based on those of previous studies. To ensure the readability of the measurement items, previous items were professionally translated into Chinese and we invited three high school students to read and give feedback on each item. Two university professors in the field of high school technology and one professor with expertise in quantitative surveys then reviewed the questionnaire for validity, and provided feedback to refine the measurement items. The questionnaires were revised three times. All items were scored on a 5-point Likert scale, with 1 meaning “*strongly disagree*,” 3 meaning “*neutral*,” and 5 meaning “*strongly agree*.”

According to Hong et al. (2021), the SROL instrument (24 items) consisted of six sub-scales, where mood management and environment structuring belong to the preparatory phase, task strategies and time management belong to the performance phase, and self-evaluation and help seeking belong to the appraisal phase. Each SROL comprised four items. For example, “Before learning online, I like to get my errands done to avoid being distracted during class” for Mood-adjustment; “Before learning online, I pay attention to whether the location is quiet for attending a lesson” for environmental structuring; “During learning online, I check the content I do not understand to ask questions” for task strategy; “During learning online, I allocate extra study time for my online courses” for time management; “After learning online, I monitor the learning effectiveness and adjust strategies or efforts” for self-evaluation; and “After learning online, I ask my classmates about the content I do not understand” for help-seeking.

Cronbach’s α was calculated to examine the internal consistency of the scale items, and Composite reliability (CR) was calculated to examine the internal stability of the scale items. The CR and Cronbach’s α values should both be higher than 0.7 to be considered as an acceptable result (Hair et al., 2019). According to the scale developed by Hong et al. (2021), the Cronbach’s α values ranged from 0.73 to 0.94 across constructs, and the CR values ranged from 0.85 to 0.95, indicating that the reliability of the SROL scale is acceptable.

### Data Analysis

First, SPSS (version 21.0) was used to calculate the Cronbach’s alpha of the SROL instrument. The Cronbach’s alpha coefficient of the SROL instrument in this study was 0.882, and the values for the sub-scales *were 0.883* (preparatory phase), 0.845 (performance phase), and 0.906 (appraisal phase). Thus, the SROL instrument had acceptable internal consistency reliability.

Second, SPSS was used for descriptive analysis of the demographic information and online course information. Then, independent sample *t*-tests were conducted to examine the gender differences in students’ SROL.

## Results

Independent sample *t*-test analysis was applied to analyze whether there were any significant differences in the variables of SROL according to gender. The results of SROL for the male (*N* = 125) and female (*N* = 275) students are shown in Table 1. The total means and standard deviations were 3.63 and 0.898 for the males and 3.92 and 0.439 for the females, respectively. A significant difference between males and females in SROL was found with *t* = –3.334, *p* = 0.001 < 0.01, and *d* = –0.410 for total SROL.

**TABLE 1:** Gender differences for self-regulated online learning (SROL).

	Gender	*N*	Mean	*SD*	*t*	*P*	*d*
Preparatory phase	Males	125	3.56	0.900	–2.702	0.008**	–0.316
	Females	275	3.79	0.500			
Performance phase	Males	125	3.66	0.916	–3.801	0.000**	–0.456
	Females	275	3.99	0.456			
Appraisal phase	Males	125	3.68	0.976	–3.120	0.002**	–0.361
	Females	275	3.96	0.499			
Total	Males	125	3.63	0.898	–3.334	0.001**	–0.410
	Females	275	3.92	0.439			

**p < *0.01.*

The means and standard deviations for the preparatory phase were 3.56 and 0.900 for males and 3.97 and 0.500 for females, respectively. A significant difference between males and females in terms of their preparatory phase was found with *t* = –0.702, *p* = 0.008 < 0.01, *d* = 0.018, where females reported better than male students. The means and standard deviations for performance were 3.66 and 0.916 for males and 3.99 and 0.456 for females, respectively. A significant difference between males and females in their performance was found with *t* = –3.801, *p* = 0.000 < 0.01, *d* = 0.456, where females reported better than male students. The means and standard deviations for self-reflection were 3.68 and 0.976 for males and 3.96 and 0.499 for females, respectively. A significant difference between males and females in the appraisal phase was found with *t* = –3.120, *p* = 0.002 < 0.01, *d* = 0.361, where females reported better than male students. In summary, the female high school students reported better than the male students in SROL during the COVID-19 lockdown. H1, H2, and H3 were thus supported. According to the mean values, the preparatory phase, performance phase, and appraisal phase were all higher for the female students.

## Discussion

There have been conflicting findings regarding the relations between gender and self-regulatory variables (Cebesoy, 2013; Martinez-Lopez et al., 2017; Park, 2019; Stanikzai, 2019). To address this inconsistency, the present study took the person-oriented approach (Alghamdi et al., 2020) to explore how the gender of individuals affected the application of SROL components in different extensions by analyzing the profiles during the COVID-19 school lockdown.

The independent sample *t*-test analysis was performed and the result showed the support of H1 as there was a gender difference in the preparatory phase, with females being more self-regulated than males. According to Hong et al. (2021) definition, the preparatory phase focuses on students’ preparatory behavior before they participate in online learning, including mood adjustment and structuring the environment before participating in online lessons during COVID-19. In the preparatory phase, the results showed that females (*M* = 3.79) had higher online SRL skills than males (*M* = 3.56). This can be attributed to the differences between females and males regarding issues that may be considered indicators of mood management and environment structuring, with females having a higher level of emotional self-regulation compared with males (Haron et al., 2010). Emotional intelligence consists of four branches: Perceiving, assimilating, understanding, and managing emotions, and females have been found to outperform males in emotional intelligence (Mayer et al., 1999). In addition, the structure of online learning environments has a clear influence on students’ readiness to join online study (Mäenpää et al., 2020). When there is a transition from face-to-face to online learning, females might adjust their methods and be more adaptable to the new learning environment (Korlat et al., 2021). Du’s (2016) study discovered that males have less tendency to structure their study environment. Thus, it can be inferred that females show higher SROL in adjusting their mood and preparing the environment to suit remote learning during the COVID-19 lockdown period.

In examining H2, the result of this study revealed that in the performance phase, females were more self-regulated than males. Pintrich (2000) highlighted that the second stage of SRL involved an active, constructive process to optimize performance strategies by effectively managing their time and effort. Hong et al. (2021) specified the performance phase focusing on task strategy and time management while participating in online lessons during the COVID-19 lockdown. In the performance phase of this study, females performed better on online SRL (*M* = 3.99) than males (*M* = 3.66), indicating that a higher level of using task strategies and managing time may be the reason why females had stronger self-regulation ability in the performance stage. The results are supported by Khiat (2019) and Wolters and Brady (2020), who pointed out that whether someone can effectively manage their time is reflected in their self-regulation capability, and Alghamdi et al. (2020) who asserted that female students tend to have more confidence in the metacognitive abilities that are necessary for employing several strategies to manage their learning and to perform tasks successfully. In addition, Ou et al. (2009) identified a time management structure that included “meeting deadlines and being organized” and “planning and using aids to manage time.” They reported that females performed better in terms of meeting deadlines and planning. Thus, with better task strategy and time management ability, females had stronger self-regulation in the performance phase than males.

In examining H3, the result of this study revealed that in the appraisal phase, females were more self-regulated than males. After learning tasks, learners then monitor and reflect on their learning effectiveness (Zimmerman, 1990; Cleary et al., 2012) and develop help-seeking schemes to improve their learning processes (Zimmerman, 2000). That is, Hong et al. (2021) specified the appraisal phase focusing on self-evaluation and help-seeking after participating in online lessons during the COVID-19 lockdown. In the appraisal phase, this study found that females performed better on online SRL (*M* = 3.96) than males (*M* = 3.68). This result is supported by Senler and Sungur-Vural (2014) and Leal et al. (2019) who suggested that appraisal may cause females to be better at self-evaluation and help seeking than males. Self-assessment skills have been shown to be important for SRL, and training in these skills has been found to significantly enhance the effectiveness of SRL (Kostons et al., 2012). Teachers should therefore guide students, especially males, to evaluate their own performance and understand their mastery of knowledge. After online learning, students often turn to their classmates and teachers for help during appraisal. Particularly, Song and Li (2020) indicated that females do better than males at seeking help online when they are supported by such online tools as search engines, emails, or question and answer forums. Females are also more likely than boys to reach out to their teachers during the COVID-19 lockdown and to develop better relationships with their teachers (Korlat et al., 2021). Learning relies heavily on interaction between students and teachers (Taylor et al., 2007), especially in the context of an epidemic. Therefore, it is suggested that teachers should take more initiative to interact with males and support their future online learning.

## Conclusion

During the COVID-19 outbreak, the trend of online learning was unstoppable in many fields including high school education. SROL involves mood management, environment structuring, task strategies, adapting strategies, time management, self-evaluation, and help seeking in the learning process. This study divided the process of SROL into three phases: The preparatory, performance, and appraisal phases. In order to understand the gender differences in high school students’ SROL, this study explored online SRL in detail in these three phases. The results show that there were gender differences in the three phases of SROL. Females scored higher on the preparatory, performance, and appraisal phases, indicating that females may be more likely to self-regulate their learning than males.

In the context of the COVID-19 lockdown, the ability of SROL has suddenly become one of the greatest challenges for students (Chen, 2020). This study attempted to provide some enlightenment for education researchers to deal with similar emergency situations in the future. High school students are likely to have a long period of online learning, and such online learning could become the norm in the future. Therefore, students’ SROL will be a hot and valuable topic. The theoretical significance of this study is to reveal that there are gender differences in the SROL of high school students in the COVID-19 lockdown. Given the paucity of studies on the three phases of SROL in high school education, this study extends the research on SRL and makes an important contribution to the empirical research regarding SROL in high school education.

### Implications

The teacher-centered instructional paradigm is the main method of knowledge transmission (Bai and Wang, 2021); as such, most students follow the lesson plans designed by teachers. SROL reminding services are better at prompting students to do more course activities and to complete learning activities than non-SROL-prompt viewers (Wong et al., 2019). Compared with male students, female students pay more attention to their learning and follow their instructors so female students are better at self-regulation than male students (Wijaya et al., 2020). Therefore, instructors should observe whether female and male students have differences in their behaviors in the three phases of SRL and give them corresponding guidance to help enhance their SROL and to ensure the quality of their online learning.

From middle school to high school, learners are potentially faced with heavier academic workloads, more stringent practice, and less guidance or supervision from instructors. The self-reported levels of SRL are also usually very low in early high school (Effeney, 2011; Barak et al., 2016). Nevertheless, students have to take online courses during the COVID-19 period, and so promoting high school students’ SROL is necessary. This study could be a reference for online course designers and online course instructors, to support them in making rational decisions regarding the promotion of online learning and the reduction of gender differences in online environments. Thus, instructors should strengthen students’ SROL, and especially that of male students, to ensure their learning effectiveness.

### Limitations and Future Study

There are some limitations of this study that should be pointed out. First, from the point of view of the source and number of samples, the participants in this study all came from the same area, and it was difficult to guarantee that these samples could represent the gender difference of SROL at all high schools. Future studies may collect representatives from more high school students of more areas. Second, the demographic information shows a predominance of female participants (68.7%), which may have resulted in skewing the distribution of the results. Future studies should therefore aim to recruit a more balanced sample in terms of gender to ensure that the conclusions of this study could be applied to a wider range of population groups. Third, as they face university entrance examinations, it is quite possible that students in different grades could also exhibit differences in their SROL. Future researchers may therefore explore the gender and grade differences in the three phases of SROL.

According to the results of this study, the use of a time management enabling system can facilitate and guide students to study in a consistent manner, and can help them to practice more effective time management. Future online learning could thus use an automated adaptive time reminding enabling system to guide male students in particular to better manage their time.

## Data Availability Statement

The original contributions presented in the study are included in the article/supplementary material, further inquiries can be directed to the corresponding author/s.

## Ethics Statement

Ethical review and approval were not required for the study on Human Participants in accordance with the Local Legislation and Institutional Requirements. Written informed consent from the high school students/participants was not required to participate in this study in accordance with the National Legislation and the Institutional Requirements.

## Author Contributions

All authors contributed equally to the conception of the idea, implementing and analyzing the experimental results, writing the manuscript, and reading and approving the final manuscript.

## Conflict of Interest

The authors declare that the research was conducted in the absence of any commercial or financial relationships that could be construed as a potential conflict of interest.

## Publisher’s Note

All claims expressed in this article are solely those of the authors and do not necessarily represent those of their affiliated organizations, or those of the publisher, the editors and the reviewers. Any product that may be evaluated in this article, or claim that may be made by its manufacturer, is not guaranteed or endorsed by the publisher.
